# Plea of Insanity—Trial for Murder

**Published:** 1859-01-01

**Authors:** 


### PLEA OF INSANITY—TRIAL FOR MURDER.

We copy from the *Times* of December 18th and 20th, the subjoined report of the trial of James Atkinson for the murder of Mary Jane Scaife. This case presents several points of interest. It is the first case within our recollection of a judge admitting, in a most liberal spirit (as evidence in favour of the prisoner), the fact of his strong hereditary predisposition to insanity. Evidence of this character is generally considered as inadmissible in criminal as well as in civil cases. We are much gratified to find that there are enlightened judges on the bench who do not close their eyes to the importance of testimony of this kind, and who are disposed to view such facts as important when considered in connexion with the analysis of obscure cases of mental disease set up as an excuse for crime.

James Atkinson, aged 24, was indicted for the wilful murder of Mary Jane Scaife, at the parish of Hampthwaite, in the West Riding of York, on the 1st of August last. The prisoner refused to plead when arraigned, and by his lordship's direction the jury were sworn to try whether he stood mute of malice or by the visitation of God. Mr. Anderson, the surgeon to the gaol, was then sworn, and stated that the prisoner, in his opinion, could both hear and perfectly understand what was said to him. After this evidence the jury found that the prisoner stood mute of malice, and by his lordship's directions a plea of "Not Guilty" was recorded for him.

The prisoner, who is a young man with dark hair, small head, and narrow, but high forehead, appeared during the trial listless and indifferent, and as though incapable of fully appreciating his awful position.

Mr. Price and Mr. West appeared for the prosecution; Mr. Bliss, Q.C., Mr. Middleton, and Mr. Maule for the defence.

Mr. Price, in stating the case to the jury, said it was impossible, after the course taken by the prisoner, to shut his eyes to the nature of the defence intended to be set up. If the facts led the jury to the conclusion that the prisoner had inflicted the wounds which had caused the death of the deceased, it would be their duty to find him guilty. The father of the deceased was a small farmer near Darley, and the prisoner was the son of a flax-spinner at Darley, and acted as his overlooker. The prisoner and the deceased had courted for several years. Shortly before this sad event there had been a gala at Bewerley Park, at which the deceased and the prisoner were, and a man named Gill, a farmer of the neighbourhood, was there and had paid the deceased some attentions which had apparently excited the prisoner's jealousy. Shortly after this, on the 1st of August, the deceased met the prisoner as she was coming from church with her sister, and he walked towards her home with her, a man named Furness walking with her sister. The prisoner and the deceased were seen to go up a lane called Stump's-lane, and the prisoner was the last person seen walking with the deceased alive. The deceased next morning was found in the ditch in this lane, with her throat cut, and dead, and the prisoner was in consequence apprehended. It appeared that early that morning the prisoner had gone into his brother's bedroom, and had made a statement that he had cut Mary Jane's throat—"the Lord have mercy upon him, he had murdered his sweetheart." On being taken into custody, he repeated this statement, and subsequently made a lengthened statement, detailing the particulars of the crime, when before the magistrates. The learned counsel stated in detail the facts that will be found stated at length in the evidence.

William Scaife, the brother of Mary Jane Scaife, sworn, stated that he resided at Darley. His father was a farmer, and resided at Darley. The prisoner had kept company with his sister for five or six years. They were sweethearts. He last saw them together at Hurtwith Chapel on Sunday, the 1st of August, about eight o'clock at night. They were going in the direction of Darley, which is about a mile off. They were coming down a lane called Niddlane, and going towards Stump's-lane, which comes out between Darley and a place called Wreaks. Stump's-lane was the nearest road to Scaife's house. A week before witness had been at a gala at Bewerley Park. His sister (the deceased) and the prisoner were both there. A Mr. Geoffry Gill was there talking to her. His sister left the gala about eight o'clock.

Cross-examined.—It was a fine summer's evening, and people were walking about. He heard no cry.

William Downs, a farmer at Darley, uncle to the deceased, also spoke to seeing the deceased and the prisoner turn up Stump's-lane on Sunday night, the 1st of August. He was courting her, and he had often seen them together. In August, 1856, she went to Manchester, and returned in September, 1857. Mr. Geoffry Gill, a farmer at Kettlesing, had paid attention to his niece. The prisoner's father had a flax-mill, and the prisoner, who was 24 years of age, was mechanical overlooker.

Richard Howard, glass and china dealer, living at Darley, knew the prisoner and the deceased. He saw them together going up Stump's-lane on the 1st of August. About twenty minutes past five o'clock next morning he was out looking after a mare in Stump's-lane, when he found the body of a woman laid in the ditch bottom. It appeared to be on the knees and fallen back in the ditch bottom. It was blood all over, and the mouth and eyes wide open. He immediately went back and alarmed his neighbours, and they returned and made it out by the dress that it was the body of Mary Jane Scaife. They went for the police, and Mr. Horton, the surgeon.

Cross-examined.—When he saw the prisoner and the deceased walking together they were "linked" together, and appeared as loving as he had ever seen them.

John Clifton, policeman, of Darley, went to the house of the prisoner's father on Monday, the 2nd of August. He had previously seen the dead body of Mary Jane Scaife. He found the prisoner in his bed-room. He charged him with feloniously murdering Mary Jane Scaife. He said "I have; I have murdered my sweetheart." I afterwards asked him where the knife was, and he said he would go along with me and show me. We crossed three fields, and he found the knife produced (a small clasp knife) in a dry well. It was open, and the large blade was bloody. On the way to Ripon Gaol he said, "I have had this on my mind for three weeks, and I told her I would murder her if she would not have me." There was no blood on his hands then.

Cross-examined.—There was a small scratch on his finger.

Thomas Atkinson, the brother of the prisoner, examined.—He lived at Darley, with his father. When the prisoner came home on the night of the 2nd of August he came into witness's room between four and five o'clock. Witness was in bed. He said, "Lord have mercy upon me, what have I done? I have murdered my sweetheart. I must have. Have I done it? I must have."

Cross-examined by Mr. Bliss.—I was asleep a few minutes after he came home before he came into my room. He was quite undressed. I had observed a change in him lately. I had seen that he was troubled with something for some weeks before this happened. He had a brother John, who had been dead nine or ten years. The prisoner was about 24. Witness was 29 years of age. He had five sisters living and one step-brother, and two older brothers than himself. Witness could read and write only middling. He looked over the mill occasionally. The prisoner worked at mechanical work at the mill, and overlooked a little. He only did simple work. The prisoner was very passionate, and he had seen him put into a rage for a very trifling cause. If ever he was checked, he got into a rage for very trifling things. He was then quite ungovernable. He had seen him fight on those occasions like a madman. He could not say much about the prisoner's behaviour to his mother, as he was then only 11 years old. The prisoner had behaved very badly to witness sometimes. If he said half a word it would derange his mind. The knife produced was his brother's. The prisoner had four watches. One was a little silver Geneva watch. Witness knew he intended to give that watch to the deceased. His brother John had been not altogether right. He also got into rages for very trifling causes.

Mr. Price objected to this evidence as collateral.

Mr. Bliss said this was the very essence of the inquiry, which was whether the prisoner was of sound mind at the time or not.

His Lordship admitted the question. It was clearly evidence in such an inquiry; what was the state of mind of the brothers and sisters of the prisoner was most important evidence to establish whether insanity was hereditary or not in his family.

Cross-examination resumed.—He had an aunt Ann, the sister of his mother. She used to get into rages in the same way as his brother for very trifling causes. She was quite insane once. She got wrong with her own mind and was put into an asylum.

Re-examined.—When his brother fought he would seize a hammer or anything. Three of his sisters were married. His eldest brother worked at the mill. He never heard that his brother had bought watches and sold them to the mill-hands.

Mr. Richard George Horton, surgeon, at Darley, was sent for on the 2nd of

August to Stump's-lane. When he got there he found the dead body of Mary Jane Scaife. He examined the body. The cause of death was the division of the windpipe with some sharp instrument. A knife like the one produced would have done it. Her dress was saturated with blood. Her bonnet was lying under her in the ditch, her parasol broken and soaked with blood.

Cross-examined.—He arrived there at six in the morning. The body had been dead several hours. There were eight distinct cuts or gashes in the throat, and two punctured wounds, one on the right cheek and one on the left clavicle. The skin was flayed off near the ear. There were three cuts across the windpipe, and the left jugular vein was severed. Several of the wounds he considered must have been given after death, as no blood had flowed from them. When the body was taken to the inn, the prisoner had desired to kiss the deceased. Witness had resided seven years in the neighbourhood. He was the medical attendant of the prisoner's family, and knew the prisoner. He should consider him a man of very weak mind. He was very readily excited if at all thwarted, and to a fearful extent. A man of decent education would not be excited by such frivolous matters as he had known the prisoner excited by. He had known him in paroxysms of passion and outrageous—almost in a frenzy. He could not say that he would be absolutely without control at such moments. He never saw the prisoner insane; but from what he had known of the prisoner's temperament there was a possibility of his becoming so. He is afflicted with goître; it is of no great size, and he had consulted witness about it. He did not know that goître and cretinism are necessarily associated together. He thought, under similar circumstances, the prisoner would do the same thing again. But possibly he would never feel the same with regard to any other girl. He had attended the deceased by the prisoner's request in March last for a miscarriage.

Re-examined.—When that connexion existed between them he could easily imagine that, if she declined to marry him, he would be so excited as to commit the crime he had done, and he believed the prisoner would do it again under similar circumstances. Violent passion might be aroused in him from various causes. Violent passion was not a symptom of weak intellect. The prisoner was a weak, frivolous, vain young man. In his paroxysms of passion he might be subjected to momentary insanity. He had been given way to so much at home, and his temper had been so little thwarted—in every argument they had given way to him so much, that he considered he was master of all, and if thwarted, momentary insanity might appear. If he had been differently educated, he probably might have been very different.

William Pullen, joiner, at Darley, saw the prisoner and deceased in Stump's-lane on August the 2nd. Witness observed to a friend, "These are close acquaintances." The prisoner said, "Not so close as some of you think."

Cross-examined.—He did not find so much fault with his sense except in this case. He was childishly fond of the deceased—never happy except with her. His conversation was not the same as other young men's. He was rather deficient. His conversation was not very amiable, and he generally repeated it over again, and laughed. He was teased for that. He seemed very passionate, and his passion was excited by mere trifles. Whenever he was checked or thwarted, he was very resolute, even about trifling things. Witness was related to the Scaifes.

Re-examined.—He had seen other young men after the deceased. The prisoner seemed jealous of her if he saw any one else talking to her. He would laugh at his own talk before anybody else would.

Mr. Price proposed to put in the prisoner's statement.

This was objected to, on the ground that it was not taken according to the provisions of the statute.

The objection was overruled, and the statement, which is as follows, was put in evidence :—

"The reason I murdered her was because she would not have me. She told me her parents would not let her have me, and she said, 'My father was not against it;' and the last words she said were, 'It's all my mother that has caused this disturbance, Jim.' She cried out, three times, 'The Lord help me ; the Lord forgive me ;' and I hope the Lord will forgive her. I told her I could not be happy without her ; I could not rest in this world. She said it was all false ; that I cared nothing about her, I had behaved so badly to her. It was all because she was queer with me made me do it. I told her I should murder her if she would not have me, because I could not rest. I told her I had not been happy for three weeks, or more than that. She told me she had not been happy since her parents were against it ; they were always calling her. Her mother was the worst, she said. Her mother never let her alone day in and day out. Her father never said anything to her, never changed words about it. I was in the house on Sunday at tea time with her. Her sister was there, and her mother came in ; her sister and her were in the house and I was in the kitchen. She came out from her mother, and said, 'Come, go out before my mother comes in.' I did as she wanted me, and we both thought she did not see me, and I went out into another house on the contrary side of the road—my father's house, but not where he lived. I should, perhaps, stop an hour there, waiting of her and her sister coming out to go to chapel. Her mother and her sister came out before her, and, perhaps, would stand talking a quarter of an hour or ten minutes, and then she came out. I went to her, and then the two sisters went up the lane and the mother went home. I followed them up the lane, and overtook them in a field that takes into Stump's-lane, where I murdered her. She did not talk to me much, but her sister did. I went down to the bottom of the lane called Stump's-lane with them, and my sweetheart told me to go home to change my coat—to get my black coat on. I said that would do that I had on for me, and we parted at the bottom of the lane. I said to myself I would go to Furniss, the other sister's sweetheart. There were some more people with Furniss. I stopped a little bit and then I walked forward. There was some more people set and standing on some wood below. I went and sat myself down, and talked a little bit, and Furniss came up, and some more with him ; and I got up after a little bit, and I went against Furniss. I asked him if he was going to Hartwith. We set off down the lane together, and he thought it was a long way to go, he said ; however, we kept moving on slowly while we got to the new line, and her brother came to us. We were set upon a wall. We talked a little bit, and I pulled out a shilling and sixpence, and asked if they could knock the shilling from under the sixpence, and the sixpence to remain on the finger end. They tried to do it, and they did it after a little bit. Then we set off all three of us to chapel. The girls had gone before we set off. When we got to the chapel gates we went forward to the public-house. We each of us got a glass of ale. Then we came down to the chapel again. Then all the people came out of the chapel. We followed after them. I went to my sweetheart, but she did not talk to me much never afterwards. She said she thought we should have to part, as they called her so. She said they had been calling her after I left the house ; her mother had near have gone crazed ; and we walked on together and left her sister and Furniss. We walked on to the Stump's-lane bottom. When we got a little way she took her arm out of mine. I wanted her to put it in again, and she would not. I told her I could not be happy without her until I married her. She thought we could not be happy—she was sure we could not be happy. She told me I should behave badly to her. I said I should not if she would marry me. I'd be content with her, but not without her. She said we had better part a little bit. I told her many times I could not part with her unless I did something with her. I told her I thought there was some one else she wanted, and I could not bide any one else to have her. She said we could both do without one another a little bit. Then I took hold of her. She was walking on the side of the road. She was all the while awkward with me, and would not go on quietly, so I stopped her where she was ; I took her by the throat, and told her I would murder her if she did not go on quietly. She said it was all false. She said I only wanted to make her believe so. Then I took her by the throat and tried to choke her. She cried out when I took her by the throat, and I thought some one would hear, and we both got up and walked on a little bit, and I pulled my knife out and showed her it. She cried out, 'Let's go home, Jim; let's go home, Jim.' I seized her and cut her throat, and she cried out, 'It's all my mother, Jim, that has caused this disturbance.' Then she cried out, 'The Lord help me,' three times, to the best of my recollection, and then she fainted away, and then I left her. I went over the wall; shut the knife and put it into my waistcoat pocket. I went up the fields and wandered about, perhaps an hour, or an hour and a half; and I thought I would go and tell her parents, but I could not go then. I came back. I thought I would go to her again, and I got a little bit down the lane. My heart failed me, and I could not go to her. Then I got over the wall again to the other field on the other side of the road, and I took the knife out of my pocket again and opened it, and put it in the wall top; then I took across the field home to a little dam there is of my father's, and washed the blood off my hands and face; then I took across the field home. When I got home, my father and them were up. I did not go into the house; I went into the shed where the waggons and carts are, and sat me down till I thought they had all gone to bed; then I went into the house. I could not eat any supper. I went to bed. I could not rest all the night. I got up in the morning and told my brother as he told you, and have nothing more to say. "JAMES ATKINSON."

This was the case for the prosecution.

Mr. Bliss then proceeded to address the jury for the prisoner. He said he had to desire, what he was quite sure the jury were ready to give him, their patient attention through this case. They had heard of the part which related to the prosecution. They and the prosecution had been informed of the nature of the defence, but they had yet had no opportunity of hearing the witnesses on the part of the prisoner who were to prove it. It was to the prisoner's case, and to the evidence to be offered for him, that he now invited their attention. He thought they would find, when they had heard the case through, that it was only to be deplored; that they were not called upon by their verdict for revenge on the prisoner, although there were grounds for measures of caution and security, and to provide against any similar occurrence for the future, upon their conviction that the prisoner was not a man who was to be treated as having that degree of understanding which was requisite to make him responsible for such a crime. They saw before them at the bar one of those objects who, for the mysterious purposes of Providence, had been sent into the world not gifted with the ordinary understanding of men—not having his intellect developed beyond that of a child of eight or nine years of age, subject to passions excited by frivolous causes, and not able to control them when in paroxysms of rage—one with a mind insensible to the character of the act which he did, and driven by a blind fury to do what he had not the understanding to prevent at the time. They had before them a young man of 24, afflicted with goître, or swelling of the neck, which was the not unusual accompaniment of the kind of insanity called "cretinism," and they would hear that he had unhappily come by this from hereditary taint of his family. They would find that family conspicuous for idiotcy and lunacy; that his brother was an idiot; his aunts were lunatics; that his father's brother was a furious lunatic; that his grandmother had brought lunacy into the family; that this malady was traceable to even more remote generations, and they would find six or seven lunatics in the family, in every generation—in the prisoner's own generation, in his father's, in his grandfather's, and even in his great-grandfather's generation. They would be told that this unhappy disease was in its nature hereditary; that its existence in father or mother or grandfather proved that it existed in the family, and that it might break out in any member of the family descended from that stock. They would find an effete and worn-out stock affected with idiotcy and lunacy on both sides of the house; and if the prisoner had not, from this cause and from defect of understanding and ability, the power to understand the consequences of his acts, he was not responsible for them. They would have more than this proved, and it was to this evidence he directed their particular attention. They would have the opinions of learned physicians of great experience and skill in these matters, who had carefully examined the prisoner, and whose opinions on such a matter were infallible, and they would tell them that the prisoner was an imbecile, incapable of appreciating the circumstances under which he was placed at this time, and insensible to the nature of the act which he had done; that he was so far below the average of human understanding and capacity that he could not be looked upon as acting according to the usual dictates of reason when placed in any situation which could disturb the poor feeble intellect which he possessed. By nature his capacity was not capable of being further developed than it had been, and he stood before them at the age of 24 with the capacity of a child of ten years of age. With a capacity so unequal to control his passions and the violence of his animal instincts, he was utterly unable at the time to resist the impulses of passion, and would to-morrow repeat this dreadful act, if placed in the same circumstances. He need not tell them if he proved this of the prisoner that he was entitled to their acquittal; for our law was not so barbarous as to exact from such an intellect that which it did exact from everybody else. Although they might hear idle statements that a man was sane enough if he could walk about and do something like other people, far be it from the jury-box, when executing justice, to apply such rules to him. It would be a cruelty and a barbarism which society had never been able to bear. Not even in the times when lunacy was so little understood that lunatics were treated with chains and whips could such a sentence ever be executed. But our law at the beginning of this century had made a very salutary provision for such cases as this. Lest such a man should be allowed to go at large who had committed such a crime as this, and that this might not influence the minds of jurors, our law had humanely provided that if a prisoner should be acquitted on the ground of his insanity, it should be so found by their verdict, and that in such a case he should never have the opportunity of repeating his offence but that he should be locked up for the remainder of his life. The law was wise and good, and they would execute it in the spirit of the law, and would take care that no man was subject to the dreadful punishment of the law attached to this crime about whose sanity they had a doubt. It was not necessary that they should believe him to be raving mad, for the word "insanity" applied to various states of the mind. It was sufficient if he was shown to be incompetent of knowing the consequences of his acts, and was so far imbecile as not to know the character of what he was doing, whether it was right or wrong. For the commission of this dreadful crime no motive had been proved. No jealousy had been proved. Nothing more had taken place to account for it than a mere lovers' quarrel. Would that in a sane mind account for such an act as this? What sane man would have acted as the prisoner had done? He had made no attempt to escape. Nothing had been denied him by his sweetheart. The intimacy had been carried on for years to the last extremity; they were walking lovingly together. It was impossible to account for the murder under such circumstances, except on the grounds suggested by the defence, that the prisoner's intellect was weak, that trifles easily inflamed him into rage, and that when in that state he was utterly insensible to the consequences of what he did. The prisoner was the eighth child of Thomas Atkinson, his father. His brother John, who was an idiot, now dead, was the ninth child. The prisoner, from early youth, had been incapable of learning more than the mere rudiments of education. He knew nothing of arithmetic or the New Testament. He was unable to do anything but the most trifling things. He had never more capacity than a child nine or ten years of age; but his passions and animal instincts had grown with his growth. His statements made after the murder indicated the state of his mind. His reasons assigned for the act were not such as could have influenced any sane man. He had given his sweetheart ten wounds, some after death. What criminal so barbarous and unrelenting that he could not be satisfied with the blood of his victim but he must inflict on her wounds after her death? If possessed of sane intellect, such a thing could not have happened. What was there in the circumstances of the case to excite such horrid bitterness and malignity? This act had been done by the poor girl's sweetheart—by him who had wished to kiss her after her death. How could such an act be done by him, unless done under one of those paroxysms of excitement and insanity to which the prisoner was said to be subject, and when he was not responsible for his acts? The prisoner had a brother who was an idiot. His mother had two sisters who were both lunatics, and one of them had died in an asylum, and the other had attempted to destroy herself. In the next generation the brother of the grandfather of the prisoner was given to violent outbursts of passion, and at last broke out raving mad. He had a daughter Susannah, who was now alive, an idiot. The grandmother of the prisoner brought lunacy into the family too. Her mother was a lunatic, and the same hereditary malady was traceable higher in her family. Here was sufficient to prove that there existed in this unhappy stock an hereditary taint which reduced them below the ordinary level of humanity. He should prove to them by the testimony of competent men that the prisoner was an imbecile, and that he was one whom to hang would be an act of barbarity and a disgrace to our laws. The learned counsel concluded an able speech by calling the following witnesses:—

Thomas Atkinson, flax-spinner, of Darley, the father of the prisoner, examined.—Is the son of the late Thomas Atkinson, and Grace his wife. His mother's maiden name was Reynaud. His first wife was called Mary Dalby. He had had nine children by his wife Mary, three dead. The prisoner was the youngest but one. The youngest was called John. He was born in 1837. He died thirteen or fourteen years ago, at ten years of age. The prisoner went to school to Snow's and Atkinson's. He could not tell how long he was at school. After he left school he turned him into the mill to job, sewing lists and oiling the machinery, and to look after the hands and see that they were at work. He never bought or sold for him. He never kept the books for him or wrote the letters. He was not so sharp as some of his children. When he was put out of the way he was very dangerous. Any trifling matter that did not agree with his mode of thinking put him out of the way. There were only five young people in the mill; he was very violent with them sometimes, and one of them, named Wilson, he used to caution, as he feared he would do him some harm. Three weeks before this happened he saw a difference in the prisoner. He got low-spirited, and seemed to have no talk. His son John was four years before he could walk, and he never could understand him properly. He was rather curious, and not right in his faculties somehow. The prisoner when crossed used very bad language, and was bewildered in his looks; he never dared cross him. On such occasions he would push about and seem to be in quite a rage. His wife had a sister called Ann Dalby. She was in an asylum once or twice in her life. She had a sister called Eleanor. She was wrong in her mind. His grandmother on the father's side was named Medley; she was before his time. His father had a younger brother named James. He had heard tell of him, but never saw him. His grandmother on the mother's side was named Dalviel.

Cross-examined.—His wife's two sisters were in Wakefield Asylum. One was dead. His son, the prisoner, had attended a night school in the winter for a year or two. His son attended at the mill, and sometimes took off a wheel when witness told him. The prisoner "brayed" his younger brother, and was cruel in his disposition. He had named him as one of his executors in his will, made two years ago.

Re-examined.—His wages were 5s. a-week.

Joseph Trees, joiner, of Darley, sworn.—He had lived at Darley all his life, and had known the prisoner. He met the prisoner on the morning of the 1st of August last—the day of the murder. He showed witness a Geneva watch—a small ladies' watch. He said he had got it from Robert Scaife, and he thought it would suit Mary Jane. He had three other watches. He saw him that night walking with the deceased. He looked as though he was in trouble. He had noticed that the prisoner was very passionate. He would throw things about in his passion.

Honan Potter examined.—He had married a sister of the prisoner, and had lived in the same house with him. He was a very passionate person. In his mind he considered him "rather short," and very dull. He got into raging passions for very trifling matters. He always considered him not right. On the Sunday of the murder he saw him; he looked in a wild state, worse than usual, and he had remarked on it when he got home. He seemed very fond of the deceased, as if he could not bide her out of his sight.

Cross-examined.—He had six children; one of them was 19. Some of them sometimes got into violent passions and for trifling causes. When in his passions the prisoner would do anything to be master. He cooled down when he was taken no notice of.

Re-examined.—The prisoner got into different passions to any he ever saw his children in.

Richard Gill examined.—Was a yarn dyer and maker-up for the prisoner's father, and had been in his service for the last eight years. He knew the deceased; she and the prisoner always seemed very loving. The prisoner used to call her his intended. He always considered the prisoner as a stupid man, very much given to passion. If crossed in the least, he was in a passion. He always considered him as a man not sound in his mind. Witness knew the prisoner's brother John. He always considered him out of his mind.

Cross-examined.—He was one of the men in the mill overlooked by the prisoner. He used if crossed to get into violent passions. He thought he would have killed his little brother once; he was nearly 16. He knocked him down and kicked him. He never tried to knock a man of his own size down. Witness never durst cross him. He had crossed witness many a time. He never attacked witness. Witness was between 60 and 70 years of age.

Re-examined.—He really was afraid the prisoner would have killed his brother once. The young one got away and got home. Witness dared not cross him because of his passion. He feared he would strike him.

Christopher Atkinson sworn.—Is no relation to the prisoner. He was formerly in the army and in the Peninsular war. He went afterwards to Shaw-mill, kept by John Atkinson, the uncle of the prisoner, and he then removed to Darley. He kept a school. The prisoner and his brother John, who was a complete idiot, attended his school. The prisoner while at witness's school was very dull, of weak mind and weak intellect. His manner was eccentric and singular. He was very passionate, and at other times quite low, and would scarcely answer a question. He had seen the prisoner frequently in a passion, and had had many complaints against him. Of late years he had also seen him in passions. He raved like a madman.

Cross-examined.—He foamed at the mouth. He would then cool down and would scarcely answer a question. He had been a schoolmaster forty years. The prisoner was by no means fit to be an overlooker.

Re-examined.—He never had a boy of so violent a temper as the prisoner. He never got master of his letters properly.

Joseph Snow, schoolmaster, examined, gave similar evidence. The prisoner, in his judgment, was not capable of consecutive thought. On one occasion he was present when the prisoner came home, and smelling a smell of cooking, he asked what there was for dinner. His mother told him. He then asked when it would be ready. His mother told him. He said, "I want some now." His mother told him it was impossible, it was not cooked. He had a stick in his hand, and he violently struck the clock with it, and his mother, to appease him, took the dinner out of the oven as it was, and gave him some. He was then eight or nine years of age. He knew nothing about the New Testament or arithmetic, or beyond *Reading made Easy*.

John Dalby examined.—Is the brother of the prisoner's mother. He had a sister Ann and Eleanor. Ann was not of sound mind. She was twice in a lunatic asylum, once twenty-five years ago for a year, and again fourteen years ago for two or three years. She attempted several times to commit suicide. She was uneasy and restless, and had delusions. His sister Eleanor was also taken to Wakefield Asylum. She died there.

Jane Dalby, the wife of the last witness, gave similar testimony.

Formal proof of the insanity of the two Dalbys and the medical certificates of their insanity were given, and also of the admission of Samuel Reynaud as a lunatic in 1824, born an idiot, with hasty temper.

Ann Atkinson, widow of James Atkinson, great-uncle of the prisoner, examined.—Her husband's mother's maiden name was Medley. She was married about 54 years ago. Her husband had been in an unsound state of mind. He studied about his soul till he got quite wrong. He jumped through a window and injured himself, and was pursued and bound, and taken care of. He was ill again in the same way several times. One of her daughters by her husband was of weak intellect, and wrong.

Isabella Reynaud, wife of John Reynaud, age 81, examined.—Her husband was brother of the prisoner's grandmother. Her husband's mother's name was Dowgill. She was out of her mind. Her husband at those times guarded her and took care of her. She shouted and sang all the night long. She knew Thomas Reynaud, her husband's brother, and he had a son named Samuel, born an idiot, who was sent to the asylum at Wakefield.

Mr. John Kitching examined.—Is a surgeon, and has had the medical management for 16 years of the Retreat, a Quaker lunatic asylum near York. He had visited the prisoner, with a view to ascertain his capacity and intellect, seven times, about an hour each visit. The result of his investigation was that the prisoner, in his opinion, was an imbecile. He meant by that a person whose mental powers had never arrived at maturity, but had stopped very far short of it. His mental powers had not gone beyond the capacity of a child of eight or nine years of age. He imputed that to arise from a very imperfect constitution, both mentally and physically. His physical constitution corresponded with his mental. His powers of observation were exceedingly low; his memory very deficient. He had scarcely any power either of reflecting or judging. The result of his examination was that, in his opinion, his moral powers had equally suffered in this very defective development. He had very confused notions of the New Testament, and of arithmetic. These examinations led him to the conclusion that he was not in mind more than a child. He considered his animal instincts and passions were disproportionably strong compared with his powers of mind, which he believed unable to control them. He was physically feeble, and had a large goître. These were appearances usually found attending an imbecile. Goître was a common accompaniment of imbecility. Insanity was hereditary. He had heard the evidence, and he had no doubt whatever the prisoner was in an unsound state of mind when he killed the deceased. He was not capable of estimating in a correct and healthy manner the nature of such an act, nor was he capable of restraining himself from the commission of any act from a consideration of the consequences.

Nor did he believe that even now he could be restrained from again committing the same act under the same circumstances.

Cross-examined.—He had on two former occasions come to give evidence of the insanity of prisoners. Once was in Dove's case. He did not say the prisoner's head was the worst he ever saw. He had asked him what a man thought with—if he thought with his heart? and the prisoner answered he thought not. He had asked the prisoner if a man could think with his legs cut off? and he said he could not tell what he would think then. He had measured his head, and said it was small. He classed him as an imperfect idiot and partial imbecile. The development of the intellect depended partly on the education and partly on the healthy condition and growth of the child. He should be of opinion that the prisoner had not been a healthy child from his appearance.

Mr. Kitching's cross-examination on abstract propositions was resumed at great length. The general result of it was that he did not believe that the prisoner at the time he committed the crime, from its attendant circumstances and from what he had heard and knew of the prisoner, was of sound mind. His animal instincts were greatly developed and beyond his control. He had heard from him that for a long period he had had sexual intercourse with the deceased. He did not know the difference between right and wrong in the sound and healthy manner of ordinary men. Witness had given evidence in Dove's case. He then propounded the opinion that if a man nourished an idea until it gained such possession of his mind as to injure his health and produce a morbid change in his brain, it then might become an uncontrollable condition of mind, impelling him to carry out the idea, and that he then would not be responsible.

Mr. Bliss objected to this reference to Dove's case, the object of which was to import prejudice into this.

Re-examined.—He had 112 patients in his establishment; 10 or 12 of them were imbeciles, and some of them could read and write and attended religious services. He had some imbeciles in his establishment who knew more of religious truths than the prisoner. There was no difference of opinion among learned men in his profession that a man might nourish an idea until it gained possession of him and became an uncontrollable condition of mind. He had learned that the prisoner's religious sentiments had been gained since his incarceration.

Dr. Caleb Williams, licentiate of the Royal College of Physicians, had been 30 years in his profession, and was visiting physician to three lunatic asylums. He had visited the prisoner for the purpose of making himself acquainted with the state of his mind several times. He thought he had very little power of reflection and very small power of judgment. His manner was peculiar. He was slow in answering questions, and his manner indicated a considerable effort of memory and understanding. He frequently said, "I don't remember," "I cannot tell," "I am not sure." His memory was very defective. Witness examined him as to his knowledge of trees and domestic animals, with which a man in his position was likely to be familiar. He appeared to possess very little knowledge of the locality where he lived. His moral faculties were very feeble. His instincts and passions were very strong. Witness should think that he had not sufficient mental and moral power to control his passions or instincts. He appeared to have a very imperfect appreciation of death. His knowledge seemed scarcely to exceed that of a child of eight or ten years of age. During a sudden outburst of passion witness believed he would have no power to appreciate the nature and quality of an act. He believed, from the evidence he had heard, and from what he knew of him, that he was incapable of appreciating the crime he had committed. Insanity was hereditary, and when it had existed in parents was very likely to show itself in children, and break out in sudden outbursts of passion. The character of the prisoner's mind was that of an imbecile—a man of defective mental power. In his opinion, the murder was perpetrated when the prisoner was in a state of violent passion, and in that condition he would have no power to restrain himself. Imbeciles were liable to violent outbursts of passion. From the opportunity he had had of judging of the prisoner, he should think he had always been an imbecile. His appearance and manner suggested the consideration whether the prisoner was feigning imbecility, and he had directed his attention to that subject, and came to the conclusion that he was not feigning. He had used every means which his skill could suggest to test the state of his mind, and he had no doubt that he was an imbecile.

Cross-examined.—He had given evidence in five criminal cases to prove the insanity of the criminals. Imbeciles were capable of some education, and therefore improved; he did not agree in the opinion that they remained always the same; they sometimes got worse. His memory would improve by exercise. The prisoner told him he had never seen a fir-tree, and did not know what a beech-tree was, and had an imperfect knowledge of domestic animals. He said he did not know a hare, and did not know what a pheasant was. He had not asked him whether he knew a cat, a dog, a horse, a cow, a donkey, or a pig. He thought the prisoner had a very imperfect sense of right and wrong. He did not believe that the prisoner would ask forgiveness of the father and mother of his victim, unless he had been urged to it. If he did it spontaneously, it would show he had some appreciation of right and wrong, but he believed the prisoner to be incapable of such appreciation. A sane man might, probably, steal, because he had not sufficient power to control his passion for money. He believed the prisoner when he committed the crime did know that he was committing the act of cutting the deceased's throat, but he did not know the nature and quality of the act; he did not know the moral nature of it—whether it was right or wrong. Weak persons, and those descended from insane ancestors, are prone to violent passion, and had not the same power of control as other persons. He had examined the prisoner in arithmetic. He did not know what 6 times 5 amounted to. He did not know how much 2 and 2 made. He should be surprised to learn that the prisoner had advanced as far as "Proportion;" if he had, it did not alter his opinion regarding him. He might have answered on the trial of Dove, that, "if a man nourished any passion until it became uncontrollable, that was moral insanity."

Re-examined.—If a man so nursed a passion it usually injured the health, affected the nervous system, and he became the subject of disease of the brain. He did not think the prisoner was capable of planning such a "murder"—it was the impulse of the moment. If he had hated the girl, he did not believe him capable of assuming to the world that he loved her. Education could not improve the prisoner beyond the range of an imbecile.

Mr. Samuel William North, surgeon, of London, and demonstrator of anatomy at York, had been nine years in practice, and was surgeon to a private asylum containing 40 patients for four years—Dunnington House, near York. He was also surgeon to the workhouse at York, where they had an average of 30 to 40 imbecile inmates. He had visited the prisoner in gaol six times, the last time last Wednesday, about an hour each time. He had applied tests to him to ascertain the state of his mind, and had arrived at the opinion that he was an imbecile. In his physical structure he had the stooping posture and bent knees common among imbecile persons. His manner of speaking was slow and hesitating, as though he had considerable difficulty in apprehending questions, and his answers were preceded by observations such as "I don't know," "happen." He did not change when speaking of the murder. His mental capacity was far below a man of his years of his class. He had the capacity of a child of 11 or 12 in his own station of society. The higher faculties of his mind were deficient. He did not consider "memory" one of the highest faculties of the mind; it was frequently possessed to a great degree by imbeciles; but the prisoner's memory was defective. He was deficient in the qualities of comparison and reflection. Witness knew the neighbourhood of Darley. There was a large wood of fir-trees there. He asked the prisoner what kind of country it was where he lived, but he could get no information from him such as would have guided a stranger, nor of the people who lived there. He put to him, "Suppose a man received 2*s.* 6*d.* a-day wages, and worked all the week, how much would he have to receive for a week's wages?" He made three or four guesses at the sum, none of which were correct. He asked several similar questions and got incorrect answers. He asked how much 1*s.* a-day would be at the end of the week, and that he answered correctly. He was asked if he knew where he was; he said, "I don't know; they tell me it is York Castle." He asked him all the particulars of the charge against him, and he seemed not in the slightest degree affected. He should say of his moral power, in the common broad acceptation of the term, he did not know right from wrong. The prisoner was examined as to his animal passions, and his answers showed that he indulged them to an inordinate extent. His experience of men of the prisoner's class was that they always indulged their animal propensities, and this was a general character of imbecility. He believed at this present moment that he was an imbecile—to use a common expression, "a natural" fool. A "natural" was a common term among the lower orders. The character of the crime and the prisoner's conduct subsequently were in keeping with his weak state of mind. He thought he had not a full and sensible knowledge of the criminality of his act. Imbeciles, while violent in their passions, were also timid. He believed when in those passions they had no complete knowledge of what they were doing, nor of controlling themselves. Any one form of insanity in an ancestor might be succeeded by a different form in the children. It was common experience. The more numerous a person's ancestors afflicted with insanity were, the more likely was he to have insanity. It was a conclusion upon which all medical men agreed.

Cross-examined.—This was the first time he had been examined in such a case. Sometimes idiots were gibing muttering fools, but rapidity of utterance certainly did not characterize all forms of insanity. He asked him if he knew Brimham Rocks, a famous place in his neighbourhood, and he could tell him nothing about it. He asked him if he knew the road to Hardcastle-garth, a village two miles from Darley, where the prisoner lived, and he said he did not know. He thought the prisoner knew that he was cutting the deceased's throat, but not that it was a crime. Although the prisoner had called this a crime, witness did not know that he knew it was one. Insane people often used sane expressions. He believed that the prisoner repeated the words by rote. In the general and common acceptation of the term he did not believe that he knew the difference between right and wrong.

Dr. Forbes Winslow examined.—Is a physician and a D.C.L. of Oxford, lives in Cavendish-square, London, and has studied medicine all his life. He had visited the prisoner for more than an hour the day before yesterday, and examined him most carefully as to the state of his mind, with a view to this inquiry. He appeared to him to have the physiognomy of a class of persons called "imbeciles." The expression was fatuous and vacant, as often seen in persons subject to *dementia*. He had the peculiar slouching gait and attitudes of that class. He has also a large goître, and knowing that that feature is often seen with a low order of intellect, particularly among a class of people in Switzerland called "cretins," and from his general aspect altogether, had he met him in a room, he should have been much struck by his imbecile appearance. He was remarkably slow and dull of apprehension. He answered in monosyllables, and as if he had difficulty in giving an answer. He had a slow, sluggish style of thought, and answered with difficulty questions which a child of five or six years of age would have readily understood. He questioned him as to his unhappy condition, and he did not appear to know where he was. He said he was told he was in York Castle. He did not know whether it was in Yorkshire; it might be in London. He asked him if he knew he had committed a dreadful crime. He said, "Parson tells me so." He asked if he was to be tried. He said "I supposed so. Parson tells I is to go before judge." He asked him as to the existence of a God. He answered "I never seed Him." He asked him if he knew who our Saviour was. He said he didn't know; parson told him about hell, and that it was fire. He did not know where it was, it might be in Yorkshire or London. He did not seem to know things which children of ordinary knowledge were acquainted with. He did not know whether the Queen was a woman or a man. He was able to repeat the Lord's Prayer correctly, at which witness was rather surprised, considering his past answers. He had seen a vast number of imbeciles, both at home and abroad. It was usual, in some conditions of sad imbecility, for patients to show a proper knowledge of the great truths of religion. They very rarely forgot the lessons of early days. Imbeciles were capable of being instructed up to a certain point, and even idiots. That experiment had been tried in the various idiotic institutions. He had never known an imbecile do a sum in "Proportion;" but one might, by careful and continuous training, be educated to that extent. It was utterly impossible for the prisoner to understand such a sum. It was a lamentable fact that just as the higher faculties of the mind sunk down, the animal passions began to master the understanding, and took the place of reason. Imbeciles often showed great affection. Indulgence in sexual intercourse would be very likely to absorb the rest of the mind. Imbeciles were constantly put to manual labour in the great establishments in the country, and were quite capable of many ordinary occupations. They constantly did it. Most of the land in connexion with our great lunatic asylums was worked by imbeciles and lunatics. The facts of the present murder were consistent with the acts of an imbecile. From his examination of the prisoner, he was in a state of imbecility, not responsible for his actions, of stunted intellect, born in that condition, and he could not bring his mind to believe that he ever could be looked upon as a healthy-minded person and responsible for his actions. He might have a physical consciousness of an act done. This was a case respecting which he had not the slightest doubt, and he never saw a case clearer to his mind than the state in which the prisoner now was.

Cross-examined.—There were cases of feigned insanity. He had seen such cases, but had not been deceived by them. He could not tell where Hell was, nor could witness. He had no idea who God was. Witness should expect a child of five or six properly instructed would be able to give some notion who God was. Cretinism was not confined to Switzerland—there was a great deal in Lancashire. Goître was not confined to lunatics—certainly not. Imbecility came on as life progressed. He did not agree with Dr. Taylor's definition. He did not agree with Dr. Taylor's opinion that one-half the world might reason itself into the belief that the other half was insane. Dr. Taylor was an eminent man, but he had not had much experience in this branch of science. An imbecile was one whose brain was stunted in its development, and as a consequence the mind was feeble, the brain being the organ of thought. He never knew of a sound-minded criminal who had covered his victim with wounds. He had in a number of cases refused to give evidence in support of a plea of insanity, and had made it a rule to refuse to go into a witness-box to prove a prisoner's sanity on a charge of murder. He had in many cases been called as a witness in support of a plea of insanity. He thought the prisoner had been in this stunted state of intellect for years, and he could not conceive that the prisoner had a right appreciation of the wrongfulness of the act he was doing when he committed the murder. (A letter written by the prisoner from the gaol was read, full of religious scraps and quotations.) The witness thought the letter showed more apprehension than he should have given the prisoner credit for. (Another letter was read.)

Mr. Bliss objected to this evidence. The defence having been disclosed by the cross-examination, these letters ought to have been tendered as evidence in chief.

Mr. Price said the plea of insanity rested on the defendant to prove, and he could not meet that defence until it was raised. The issue he had to prove was the murder.

Mr. Bliss, in reply, said his friend was perfectly right if he had not known, from the defence set up by the cross-examination, that insanity was the defence. He now changed the case. He went partly into the case before, and now again went into the other part.

His Lordship was of opinion that the evidence was admissible by way of reply.

Examination continued.—If the letter read was a spontaneous act, and the prisoner had not been directed to write it, it exhibited more power of mind than he should have supposed the prisoner possessed. (A third letter was read from the prisoner while in gaol.) If spontaneous, the prisoner seemed to have more appreciation of his position than he should have imagined.

Re-examined.—It was possible for an imbecile visited by a religious man or clergyman to express himself in the terms of his visitor for a short time. Unless he had been very much deceived, the prisoner could not retain such ideas long. There was nothing in his conversation with the prisoner on religious matters corresponding in the slightest degree with those letters. He should not for a moment have believed him capable of spontaneously writing such letters. He had not a shadow of a doubt as to his condition. He might be taught a few Scriptural phrases. He was not feigning insanity when he saw him. Many imbeciles were capable of writing long and kind letters. He had known many such instances. Taken by themselves, the letters were no safe test. He had taken for his conclusions the whole case together.

Mr. North was recalled.—The prisoner's was not a case of feigned imbecility.

This was the case for the prisoner.

Mr. Price called evidence in reply, proving the prisoner's letters.

Mr. Jackman, who taught the prisoner at a night school, was called to prove that he had advanced in arithmetic as far as "Proportion."

Mr. Anderson, the surgeon of the York Gaol, was called, and proved visiting the prisoner in his capacity as officer of the prison. In his opinion the prisoner was sane, and answered the questions put to him like a sane man of his class in life.

Cross-examined.—Some four or five medical men in York had devoted their lives to the study of lunacy. He did not think that those gentlemen were better able to give an opinion on questions of lunacy than himself. Such gentlemen, from devoting themselves exclusively to one subject, were liable to be a little crotchety.

Mr. Green, Under-Governor of the gaol, stated that the prisoner had written letters in the common wardroom, and he believed he had not written any letter under dictation or in the presence of the chaplain.

Cross-examined.—During the last six weeks the Wesleyan minister had visited him twice a week. He was not present. There were no writing materials in the room where they were.

Mr. Bliss then addressed the jury in reply to this evidence with great force. The learned counsel denounced the attempt which this last evidence showed to hunt an idiot to the scaffold. He knew the excitement when a verdict was at stake, and that when men got their blood up in such a pursuit, there was no fox-hunting equal to man-hunting. He denounced the attempt to sneer down the evidence of men like Dr. Forbes Winslow, Dr. Williams, and Mr. Kitching and Mr. North, who had devoted themselves to this branch of study, by the presumption and ignorance of men like Mr. Anderson, with his limited experience of York gaol. And for what? In order that this man might be hanged, and that an imbecile might be strangled on the gallows—an act of barbarity sufficient to make the very stones of York cry out against it. He called on the jury to attach no weight to evidence like this when contrasted with the mass of evidence he had adduced contradicting it. This was a question of medical testimony altogether. Which did they believe? Mr. Anderson, or the medical testimony opposed to it. The letters of the prisoner put in evidence, he submitted, established rather the imbecility which was said to be the character of the prisoner. There was in them an attempt to deal with ideas newly acquired, and a repetition of the same scriptural quotations, sometimes misquoted, which indicated imbecility; and they were told that letters alone were no test of sanity, but that imbeciles were often able to write proper letters. The letters were, he suggested, the promptings of the Wesleyan minister who had visited the prisoner two or three times a-week, and had been written from his promptings, with infinite labour. That Wesleyan minister the prosecution had not dared to call. When examined as to "hell," his answers were, "Parson says it's so and so." That was the key to the letters. He had been prompted and urged to write them. Was it upon a conflict of testimony like this that this man was to be hanged, against the opinion of the men he had called? The execution of such a man would be a disgrace to the jurisprudence of this country.

Mr. Price replied at considerable length on the whole evidence, contending that the facts showed the prisoner to be sane. Feeling the greatest respect for medical testimony supported by facts, he called on the jury to reject the testimony of a set of hired advocates in that profession, who went about to prove the theories of their own crotchety brains. The learned counsel then went with great minuteness and detail through the evidence in an able address, which occupied upwards of two hours and a-half in the delivery.

### THE SUMMING UP OF THE JUDGE.

The Court was again thronged this morning, but was not so crowded as during the two previous days of the trial. The prisoner, whose conduct throughout the trial strangely contrasted with that exhibited by him for some days after the murder, did not appear to have suffered much mental anxiety, and during the summing-up, as on the preceding days, he maintained a degree of impassiveness which was painful to witness, and which was only explicable on the assumption that he was unconscious of what was passing around him, or was acting a part with the most consummate skill.

His Lordship said, the prisoner was indicted for the wilful murder of Mary Jane Scaife, on the 1st of August last, and the jury had two questions to decide, first, whether, supposing the prisoner to be of sound mind, he was guilty of the murder; and, second, whether he was of sound mind at the time he committed it. The plea set up by his learned counsel was, that at the time he committed the crime he was not a person capable of governing his actions—that he was in a state of mental insanity, and was, therefore, not responsible. They had had a very long inquiry, and he must say that one-half the time would have done justice to every person, for, on looking at his notes, he found questions asked over and over again, the only object of which could be to embarrass their minds as to the conclusion to which they must come. The real question for their decision was the state of the prisoner's mind at the time he committed the offence. It was most important that all persons who were guilty of great crimes should be punished; but whilst it was important that the guilty should not go unpunished, both humanity and justice demanded that they should closely investigate a plea of insanity, so that they might not be guilty of the barbarity of sending a man, irresponsible for his actions, to the gallows. The case, as he had previously stated, had lasted twenty-and-a-half hours, of which five had been consumed by speeches of counsel. When the wise and salutary rule was laid down with reference to the speeches of counsel, which was now the practice, it was prognosticated that their criminal courts would be made, as they had seen the previous night, the arena for display—the arena in which to struggle for victory rather than for truth—and he put it to them, whether some of the remarks which had been made were not those of mere advocates, rather than of gentlemen whose duty it was to instruct the jury, and aid them in coming to a just conclusion. It was indifferent to himself or to the jury whether the prisoner was found guilty or not, provided they sifted the case, and came, in the exercise of their best judgment, to a true conclusion. Truth was their object; and it should be remembered that this was not a Nisi Prius cause, where every point was to be fought to the last, but a case of the gravest importance, involving no less an issue than the life of the prisoner at the bar. To proceed, they must first decide whether this was a murder, and next, if the prisoner was of sane mind. There could be no doubt as to the first question, for they had not only the evidence of several witnesses, but the prisoner's own confession. That confession would leave no doubt that at the time he made it he was a person responsible for his acts, and knew that he had committed the crime of murder; but, as they would remember, the learned counsel for the defence made no point of that, his case being, that at the time the murder was committed the prisoner was not master of his actions—that he was then insane, and unable to distinguish between right and wrong. The plea of insanity was a very general term, and included delusion, under which a person might be able to transact ordinary business; raving madness, idiotcy, and imbecility, and all classes of mental disease where a person could not distinguish between right and wrong. The question for their decision, to establish the prisoner's insanity, was, that at the time he committed this act he did not know the difference between right and wrong—that he was not in a state of mind to know that he was doing wrong. That was the definition of the law as laid down in the House of Lords, and by learned judges—in other words, as they had heard, whether the prisoner knew the nature and quality of the act, that he knew it was a wrong act. If he did, then he was responsible; if, on the other hand, he had been visited by the Almighty, and had been born into the world with a mind not capable of such a distinction, he was irresponsible, and they were bound to find him not guilty, leaving him in his Lordship's hands for future detention and security. The mere fact that he committed an outrageous act was no evidence of insanity, and he must deprecate any such idea as that stated by Mr. Kitching, if he understood him correctly, that a person of sound mind could not have committed this murder. Do not be carried away with any such notion, because it would be giving impunity to every person committing a great crime, as it would lead to the doctrine, the greater the crime, the greater the chance of escaping unpunished. He must also warn them as to the passion of a man. If a sane man flew into a passion, and murdered a fellow-creature, he was clearly responsible, because such a man was bound to control his feelings, and keep his passions in check. But, if a man was so diseased in his mind that his passion overpowered his reason, and incapacitated him from knowing that he was doing wrong, then he was not responsible. They had had a long argument, and the learned counsel for the prosecution had cross-examined the witnesses with great ability, but he had never struggled with the real strength of the case. The case for the prisoner rested upon his early character, his conduct at the time of the murder, and the influence likely to have been exerted upon him by his ancestors, through hereditary descent. As to his early history, the account generally given was, that he was a person of weak intellect, a simple person, and liable to fly into paroxysms of passion. They had two or three witnesses for the prosecution, and four for the defence, who spoke to this point. It might be said that they were near relatives, but who, their evidence being received with due care, so well able to know the prisoner's habits and disposition? They all said he was subject to paroxysms of frenzy; and upon that they were told, what they knew, that persons of imbecile intellect were men of strong passions, and liable to paroxysms of rage. Two of the medical men had examined the prisoner six times, another seven, and Dr. Winslow had examined him once. They were all well-acquainted with the subject of insanity, and they all held him to be of a class of insanity called imbecility. Their distinction between idiotcy and imbecility was this. Idiotcy, they said, was that state in which a person was born bereft of reason; imbecility, that state in which a person was born with weak powers of mind, and which could only be developed within a limited extent. Imbeciles, however, could do many ordinary kinds of work, as might be seen in most lunatic asylums. There was another class of evidence well worthy their consideration. It was proved that insanity in all shapes was hereditary. It did not follow that a man was insane because his grandfather or grandmother was insane, but it was said in this case, that such a fact was corroborative—that where insanity existed in former generations, they might expect to find it in subsequent generations, and the doctors told them that this was a link in the chain, but only a link. Without knowing the early history of the prisoner, it would have been weak, indeed a mere matter of opinion; but with that knowledge it was most important. Now, in the whole of the three hours' reply of the learned counsel for the prosecution, he never addressed himself to the combined evidence of these three steps in the case—that he was a person of weak mind, subject to paroxysms, that his conduct at the time of the murder was extraordinary, and that his family had been subject to the dreadful affliction of insanity. This was the mode in which the jury must look at the case, and it was for their consideration entirely—a matter requiring serious consideration. It might be said that two of the medical gentlemen belonged to a sect, worthy of all respect, but who entertained a strong opinion as to capital punishments, and were disposed to find persons guilty of capital offences insane, but they must not permit themselves to cast such an imputation on those gentlemen, unless upon very strong grounds. On the part of the prosecution, seven witnesses were called, four of whom spoke to the circumstance of the murder, and three to the early life of the prisoner. His brother John spoke to his confession of the murder, and stated on cross-examination that he had observed a change in him for some weeks, which was fully accordant with the prisoner's own statement that he had had the murder on his mind for three weeks. As to that fact, it would be for them to say whether it was a diseased mind pondering on something to be accomplished, or a sane mind brooding with malice over the commission of a crime. The brother spoke of him as being able to do only simple work, as getting into a rage from trifling causes, and as fighting and looking like a madman. He also proved that he had had the knife above a year, which showed that it had not been got for the purpose. Mr. Horton, the surgeon attending the prisoner's family, after referring to the *post mortem* examination of the deceased, said he was a person of weak mind, readily excited by trifles to a fearful extent. Mr. Horton also told them that he had a goître, and he might as well tell the jury to dismiss that point from their consideration at once, because there were many very clever, sharp people who were afflicted with the goître, and its connexion with cretinism seemed to be only a matter of opinion. On re-examination, the witness said the prisoner was a weak, vain, frivolous young man, and likely to fall into fits of momentary interesting insanity, though he had never seen him in that state. The witness Pullan also spoke of him as having a foolish idiotic laugh, and easily excited to violence. Here were three persons who spoke of him as of weak mind and subject to violent passion. Then there were the circumstances of the murder. On the one hand, it was maintained that they proved premeditation and malice, the motive of jealousy being suggested; and on the other, it was argued that the prisoner flew into a paroxysm of frenzy, depriving him of his reason, and that he then committed the act. It was for the jury to decide, and in doing so they must take the surrounding circumstances into consideration, and judge how far it was consistent with the prisoner's imbecility that he afterwards sobered down to a state of consciousness, so as to make the statement he did before the magistrates. He now came to the evidence on behalf of the prisoner. Four of those witnesses were connected with the prisoner's family, and though that might be a subject requiring their attention, they were better qualified to form an opinion than those who only saw him since November last, whilst in gaol, and when he might be said to be acting. The father, who gave his evidence very fairly, without exaggerating anything, said he had no control in the mill, neither bought nor sold anything, could not keep the books, was not so sharp as other children, and was very violent when excited. He also gave evidence as to the insanity of two of his sisters. As to the prisoner working in the mill, they had the evidence of Dr. Winslow that it was a common thing in the Continental asylums to set imbeciles to even more difficult work than that in which the prisoner was engaged. On cross-examination he admitted that he had made a will in which he had appointed the prisoner one of his executors. That was a fact worthy of their attention. It appeared inconsistent with his other statement that he did not think him so sharp as other children. It was probable, however, that the thought never crossed the father's mind. Joseph Trees spoke of him as being passionate and resolute; and Horar Potter, who married his sister, and had lived with him, said he was passionate and rageous in trifling matters, and he never considered him quite right. Richard Gill also spoke of him as not of sound mind. He had already told them that if a man of sound mind committed a murder in a passion he was responsible, but if a person of weak mind were overcome with passion, and did not know whether what he was doing was right or wrong, then he would not be responsible. The schoolmasters, Atkinson and Snow, spoke of him as a dull, passionate child at school, who got into frenzy, and foamed at the mouth for trifling causes. Then they had the four medical men—men eminent in their profession. They gave their opinions, and the jury must consider what opportunity they had for forming the opinions they expressed. Scientific men no doubt did differ very materially as to their evidence in courts of law, and the only test which could be applied was the means they had of forming their opinion. In this case, the four witnesses called had been long connected with the treatment of insanity, and, as they had heard, paid frequent visits to the prisoner in gaol, asking him some six or seven hundred questions. The result was that they all agreed in describing him as an imbecile, and they told them that it was a characteristic of imbeciles to be subject to violent passions, which overcame what little reason they had. Mr. Anderson had been much abused for giving an opinion, which was forced from him by Mr. Bliss, and for saying that men who devoted their whole time to one study were apt to become crotchety. There was no doubt of that; but they must not reject the opinions of eminent men for that reason alone. He should only direct their attention to the evidence of Dr. Williams and Dr Winslow, as the others agreed with them on the main points. They both spoke of the prisoner's physical appearance as being striking. Dr. Williams said he had but little power of reflection and small power of judging. His moral faculties were also feeble, and his instincts and passions were so strong that he would not have the power of controlling them. The witness stated that he did not think he could appreciate the nature and character of the offence he committed; and in reply to questions, he said he had examined the prisoner with the view of testing whether he was feigning or not, and he was of opinion that such was not the case. Dr. Williams was asked whether hatred, revenge, and jealousy were not the predominating passions which led to crime, and no doubt they were, but would not jealousy operate as much upon a diseased mind as upon a sane mind? Dr. Williams had no doubt as to his being an imbecile, and irresponsible for his acts. The evidence of Dr. Winslow was even stronger. He had only had one opportunity of examining the prisoner, but he declared it to be the worst case he had ever seen,—that his physical appearance was such that it would have excited his observation anywhere, whilst his mind was only that of a child five or six years of age. He said that he might be taught to read and write, and to do sums in arithmetic, but he was sure that the prisoner could not do a sum in Proportion. The prisoner's grosser passions were strongly developed, and he had not the slightest doubt that he was an irresponsible being. On cross-examination, he said he did not think this was a case of feigning; but admitted that he should be surprised if the prisoner could do a rule of Proportion, and that he must have been grossly deceived if the prisoner could spontaneously write such letters as some which were read to him. The letters showed more apprehension of right and wrong than he gave him credit for, and he did not think he was capable of writing such letters spontaneously. He might write them if prompted or under pressure. He had known imbeciles write such letters, and it would not alter his opinion unless he knew the circumstances under which these letters were written. Here were four witnesses, all eminent in their profession, who, whilst differing upon some points, agreed in considering the prisoner an imbecile, and as being incapable of appreciating right from wrong. In addition to this, they had evidence as to six or seven of his ancestors, both on the father and mother's side, and of one brother, who were insane. That was the case for the prisoner. The previous night three letters from the prisoner had been put in. They contained many most pious observations, and he could not detect in them any wandering of mind. They were written free from excitement, but, though quoting a beautiful passage from Isaiah (which in one form or other was in them all), and speaking of repentance and redemption, they did not develop anything to show that the prisoner knew at the time what he was writing about. The doctors told them that imbeciles did write long and correct letters, showing a just appreciation of religious truth, and unless they knew the circumstances under which these were written, their opinion would not be altered. The prisoner, it appeared, had been attended by the chaplain, by the weekly lecturer (Mr. Stewart), and by a Wesleyan minister, and it was suggested that these letters might have been written under their suggestion, and that the prisoner merely used the passages and Scripture terms impressed by them upon his mind. After expressing his conviction that the obligation to call these gentlemen as witnesses rested with the prosecution, his Lordship proceeded to refer to the evidence of Mr. Anderson. That gentleman had done nothing more than his duty in attending there. Mr. Anderson had seen the prisoner three times a week since he came to the Castle, and thought him sane, but he had not tested him on a variety of subjects, and for that length of time which the other medical men had done. His Lordship also referred to the evidence of Mr. Griesbach, the schoolmaster at the Castle, and to that of Mr. Jackman, as to the prisoner getting to the rule of Proportion in arithmetic, and remarked that they had no evidence to show under what circumstances he did so. Such, said his Lordship in conclusion, were the facts. If they thought that the prisoner at the time he committed the act knew the distinction between right and wrong—that the act was a wrong one, they must find him guilty; if they thought he did not know that the act was a wrong one, they must acquit him on the ground of insanity, and he had then the power to commit him to an asylum. He had gone over the whole of the case with care, and it was for the jury to return such a verdict as in the exercise of their best judgment they thought ought to be given.

The jury retired at five minutes past eleven, and after they had been absent three hours and a half,

Mr. Bliss reminded his lordship that he had not directed the jury, in case of doubt, to give the prisoner the benefit of it.

His lordship replied that he had given the jury such directions as he thought were necessary.

Mr. Bliss pressed the point, upon which

His lordship said:—I did not direct them specifically upon that; but if I had said more, I might have said something much more injurious to your client.

The jury returned into court at ten minutes past three.

In reply to the Clerk of Arraigns, the foreman first gave a verdict of "Guilty," upon which some of the jurors called out, "Not guilty;" and he then corrected himself and said, "We find the prisoner NOT GUILTY, on the ground of Insanity."

The prisoner was then ordered to stand down by his lordship. It was difficult to observe any change in him; but his cheek seemed somewhat blanched, and his eye struck us as brightening up as soon as the verdict was returned.

---

The following article upon the foregoing trial, from the leading newspaper of the district in which the murder occurred, and the accompanying letter upon criminal responsibility, addressed to the editor of the *Times*, are worthy of being quoted.

THE DARLEY MURDER.—The acquittal, on the ground of insanity, of the wretched creature who was tried last week at York for the murder of his sweetheart at Darley will, we believe, be received with general satisfaction. No one can read the evidence without coming to the conclusion that Atkinson's intellect is originally of the very lowest type, and that such as it is, it has received an exceedingly small amount of education, either directly or from circumstances. At the same time, while his mind was thus stunted, his passions appear to have been placed under little restraint by those around him, and the rule both at home and in the factory was to let him have his own way in everything. It seems to have been generally understood at Darley that the prisoner was weak and foolish, although, perhaps, no one would have ventured to call him either an idiot or a madman. We are assured by those who had the opportunity of seeing him at York, and who were, in the first instance, rather prejudiced against the defence set up, that his appearance was quite that of an imbecile. When the history of the prisoner's family was given to the Court, there was quite enough to explain any amount of insanity in himself. His younger brother, the next in age to himself, was an idiot, and both on his father's and mother's side such a fearful amount of idiotcy and insanity was proved as it is perfectly shocking to contemplate. The medical men examined for the defence, who are persons of standing and reputation in their profession, agreed in regarding the prisoner as an imbecile whose mind was hardly more developed than that of a sane child of eight or nine years of age, and stated their belief that he could not correctly appreciate the moral nature of his acts. We are quite disposed to admit that we cannot go so far as some of these gentlemen in their definition of insanity; but the question in this case is, not whether on some subjects Dr. Winslow and his fellow-witnesses hold mistaken views, but whether they had sufficient grounds for thinking Atkinson an imbecile. Those grounds they stated to the Court, and taking them in connexion with the whole of the prisoner's previous history, with the opinion which his neighbours had formed about him, with his striking personal appearance, and with the melancholy annals of his family, there can be little doubt that the jury came to a proper decision. It must be remembered that the verdict of acquittal on the ground of insanity in criminal cases amounts virtually to a sentence of imprisonment for life among criminal lunatics—a fate not much less appalling to contemplate than death on the gallows. These acquittals on the ground of insanity are not likely, therefore, to increase crime; for no sane man would be induced to commit murder by the hope that he might possibly by a plea of insanity be consigned to a lunatic asylum for life instead of to the gibbet.—*Leeds Mercury*.

### CRIMINAL RESPONSIBILITY.

*To the Editor of the Times.*

Sir,—In *The Times* of Saturday last there is the record of a trial at York, which has a terrible interest alike for the physician, the statesman, and the public. I write in ignorance of the verdict which will be pronounced upon the unhappy man, James Atkinson, tried on the Northern Circuit for the murder of his sweetheart. Before this letter can reach you, however, that verdict will have been spoken, and will consign the prisoner either to the condemned cell of the gaol or to the wards of an asylum for criminal lunatics. Of the fact of the murder there is no doubt. The motives, too, such as they are, seem plain enough. It is the old, old story—passion and pride, jealousy and affection, the strongest emotions of the mind and the most untameable lusts of the flesh doing the devil's work in a weak, untaught, unfurnished, irascible, brutal nature. The whole essence of the case is contained in the prisoner's declaration, which is an awfully simple and natural exposition of one of the lowest phases of humanity. He had threatened to murder the girl if she would not have him. "I told her," he says, "I could not be happy without her; I could not rest in this world. . . . . I told her I should murder her if she would not have me, because I could not rest." He had frequently been known to be in similar paroxysms of rage; the girl herself, probably, was accustomed to them—"he had treated her so badly," she told him. According to other witnesses, the slightest cause sufficed in this poor wretch to bring on an ungovernable fit of passion. When in this state he foamed at the mouth, and lost for the time all self-control; when he cooled down, he would not speak to any one. He had once nearly killed his younger brother. "He knocked him down and kicked him; he never tried to knock a man of his own size down." This vicious temper was quite apparent even when he was at school; a marked instance of it occurred when he was eight or nine years of age. According to his teachers, moreover, he was at that period "very dull, of weak mind and weak intellect," . . "not capable of consecutive thought." He appears to have made very little progress even in the most elementary education; and although his father employed him in his business as a kind of overseer of the workmen, he never could be trusted with buying and selling. One of the witnesses considered him "rather short," and "very dull." Another thought him "a stupid man, very much given to passion;" and further, had "always considered him a man not sound in his mind." His brother had been an idiot, and died at ten years of age. He himself had a goitre. His two maternal aunts were insane. Several of the father's relatives also had been of unsound mind. That is the whole story in so far as it bears on the question of criminal responsibility. It is impossible by commentary or argument to make it any clearer.

My object in writing you now is, not to anticipate the decision of the jury as to whether this fearful tragedy was the result of an insane impulse or not. Take either case. Suppose it to be ruled that this young man was not insane outright, and that his crime was that of a coward and a bully, turned into a wild beast for the time under the influence of jealousy and lust. The picture is repulsive enough, and in any case it is not unlike the object. Still, can it be fairly said that society has done its duty towards this dangerous being? His tendencies were well known, and had often produced outrages which made him a terror to his family and to the neighbours. Were there no means of bringing this moral nuisance under the notice of the authorities at a point short of murder? and if brought into notice, were there no means by which it could be controlled in time?

Suppose, on the other hand, that the jury declare the act to be that of a madman, will any one doubt that the protection now to be afforded was really due long before? Was it necessary that this unfortunate man should cut a throat in order to prove himself insane? Or would it not have been at once humane and just, not to say politic and expedient, to try this question long before the murder; say when he knocked down his imbecile brother and kicked him within an inch of his life!

These questions are, perhaps, more easily asked than answered. But they are of immense importance to the public, and often present themselves in an embarrassing form to those who, like physicians and magistrates, have to deal with unhealthy and vicious natures. The medical man of the family in this case did not see it to be his duty—probably he was never asked—to interfere. I cannot wonder at it, for it is probable he could only have interfered at the risk of his own life, or, if he had placed the patient in an asylum, at the risk of an action for false imprisonment. But more and more every day we who practise the healing art are led to consider what is to be done with those who, from hereditary or acquired mental deformities, may be said to be ever on the verge of insanity or crime, even if they have committed no overt act. Must the drunkard drink himself into *delirium tremens*, and how often, before he can safely be set down as insane? Must he utterly ruin his family, perhaps attempt his own life, perhaps murder his nearest of kin, as was done the other day at the Bridge of Ean, and as is recorded in one case in the very paper which contains the trial of James Atkinson? Must the low, disgusting, selfish, corrupting elements be allowed to fester in the breast of an irreclaimable savage, until they break out into acts of the nature recorded in this and many other cases? Or will the opinion of society—will your powerful pen, for instance—justify the interference of the law to protect those who are clearly unable to control themselves, whether they are technically and in the eye of the law insane or not? Doctors and magistrates could do much in this direction; but they cannot act unless supported by the law, and by that public opinion which gives its application in particular cases.

I am, &c.,

Edinburgh, Dec. 20. A PHYSICIAN.

